# Evaluating nanobiomaterial-induced DNA strand breaks using the alkaline comet assay

**DOI:** 10.1007/s13346-022-01178-7

**Published:** 2022-05-25

**Authors:** Melissa Anne Tutty, Gabriele Vella, Antje Vennemann, Martin Wiemann, Adriele Prina-Mello

**Affiliations:** 1 Nanomedicine and Molecular Imaging Group, Clinical Medicine, Trinity Translational Medicine Institute (TTMI), Trinity College Dublin, St James’s Hospital, Dublin 8, Ireland; 2 Laboratory for Biological Characterization of Advanced Materials (LBCAM), TTMI, Trinity College Dublin, St James’s Hospital, Dublin 8, Ireland; 3 IBE R&D Institute for Lung Health gGmbH, Münster, Germany; 4 Trinity St James’s Cancer Institute, Trinity College Dublin, St James’s Hospital, Dublin 8, Ireland

**Keywords:** Nanobiomaterials, Genotoxicity, Comet assay, DNA damage, HepG2, Liposome, AuNP, PACA, LipImage™815

## Abstract

Due to their unique chemical and physical properties, nanobiomaterials (NBMs) are extensively studied for applications in medicine and drug delivery. Despite these exciting properties, their small sizes also make them susceptible to toxicity. Whilst nanomaterial immunotoxicity and cytotoxicity are studied in great depth, there is still limited data on their potential genotoxicity or ability to cause DNA damage. In the past years, new medical device regulations, which came into place in 2020, were developed, which require the assessment of long-term NBM exposure; therefore, in recent years, increased attention is being paid to genotoxicity screening of these materials. In this article, and through an interlaboratory comparison (ILC) study conducted within the Horizon 2020 REFINE project, we assess five different NBM formulations, each with different uses, namely, a bio-persistent gold nanoparticle (AuNP), an IR-780 dye-loaded liposome which is used in deep tissue imaging (LipImage™815), an unloaded PACA polymeric nanoparticle used as a drug delivery system (PACA), and two loaded PACA NBMs, i.e. the cabazitaxel drug-loaded PACA (CBZ-PACA) and the NR668 dye-loaded PACA (NR668 PACA) for their potential to cause DNA strand breaks using the alkaline comet assay and discuss the current state of genotoxicity testing for nanomaterials. We have found through our interlaboratory comparison that the alkaline comet assay can be suitably applied to the pre-clinical assessment of NBMs, as a reproducible and repeatable methodology for assessing NBM-induced DNA damage.

**Graphical abstract:**

Workflow for assessing the applicability of the alkaline comet assay to determine nanobiomaterial (NBM)-induced DNA strand breaks, through an interlaboratory comparison study (ILC)

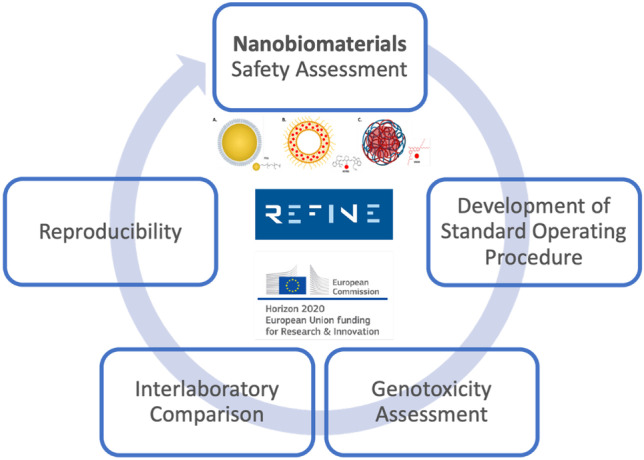

**Supplementary information:**

The online version contains supplementary material available at 10.1007/s13346-022-01178-7.

## Introduction

Nanobiomaterials (NBMs) are currently at the cutting edge of the rapidly developing field of nanotechnology [1], from adaptive manufacturing (e.g. 3D printing and self-assembly) [2] to cosmetics and food (e.g. liposome, micelles) [3–5] to medical applications (e.g. pacemakers, prosthesis, dental fillers, and nanomedicines) [6–10]. However, despite their promise and increasing number of applications, there is still great concern regarding the potential toxic effects and unpredictable health outcomes associated with them, and the hazardous effect they may have on human health and the environment [11–14].

Decades of toxicology research applied to nanoproducts (i.e. nanoparticles, nanocoating, nanofillers, nanofibers) not only has shown the complex interactions between nanomaterials and cells, humans and the environment, but has also presented a knowledge gap, where more in-depth work is needed to fully distinguish on how the physicochemical characteristics of NBMs applied to the specific application influence the interactions. This can be defined as a purpose-specific assessment. Generally, risk assessments are carried out for NBMs; however, their unique characteristics often cause unpredictable outcomes. Therefore, there is a great need for more comprehensive information on the physicochemical properties of varying NBMs and on how they behave in different environments or biological systems [15, 16].

There is a wealth of research that has been reported for both in vitro and in vivo toxicity screening performed on engineered nanoparticles [16, 17]. As nanomaterials can enter the cell and interact with cell components and can also persist in the body causing chronic toxicity, in recent years, more importance has been placed on their toxicology testing [16]. However, despite the barrage of screening techniques available for cytotoxicity and immunotoxicity screening, somewhat less attention is placed on the screening of nanomaterials for their potential to alter the genetic information of a cell, i.e. genotoxicity. As nanomaterials can enter the cell nucleus, either intentionally or unintentionally, they interact with DNA causing DNA strand breaks, damage to chromosomes, or altered bases. As these changes, which can occur at even relatively low exposure levels, are associated with such serious adverse health effects [18, 19], there is a great need to assess nanomaterial genotoxicity in an accurate and robust manner [20, 21]. Genotoxicity screening represents a vital action in safety assessment of NBMs and is also required under the European REACH (Registration, Evaluation, Authorization and Restriction of Chemicals) framework [22]. A variety of in vitro screening methods for detecting genotoxicity exist, including the bacterial Ames test; the mammalian cell gene mutation test; assays such as the micronucleus assay, chromosomal aberration test, fluorescence in situ hybridisation (FISH), and chromosome painting; and assays which measure DNA strand breaks, including alkaline elution or unwinding, and the focus of this study, the alkaline comet assay [21]. Current guidelines suggest the in vitro bacterial gene mutation test (i.e. Ames test, OECD 471), an in vitro mammalian cell gene mutation test (e.g. HPRT assay, TK test; OECD 476), and an in vitro mammalian micronucleus assay or chromosome aberration test (OECD 487 and OECD 473) [23]; however, in order to reduce false-positive results and in turn reduce subsequent in vivo screening, it has been suggested that the in vitro regime for testing materials requires adjustment. To date, much debate still exists as to the best regime for in vitro testing and minimising in vivo animal experimentation, even though regulatory requirements for chemical compounds exist clearly. However, in contrast to this, the genotoxicity screening methodologies for NBMs are far less well defined, and the best testing strategy to employ are still relatively unknown. Despite the assays available and the guidelines laid down, a standardised genotoxicity assay for NBMs does not yet exist [24], and whilst a vast array of information exists on the genotoxic potential of various chemicals, limited information exists on medicines and medical devices due to the fact that they have only been introduced into toxicology and long-term screening in the last 5 years. This is in part due to the use of inorganic materials as carriers.

Each of the genotoxicity assays previously mentioned has their own intrinsic advantages and disadvantages. Whilst the Ames test is well described as the most reliable genotoxicity screening method, it is a poor predictor of large-scale DNA damage and is often cited as unsuitable for assessing NBMs [25]. The in vitro micronucleus assay, which determines the frequency of gross chromosomal damages induced by compounds and NBMs, has become more popular compared to the chromosome aberration assay as it is easier to undertake and quicker to perform and analyse, as well as its ability to detect clastogens (something the chromosome aberration assay cannot do unless modified). Despite being recommended as a core in vitro assay for characterising genotoxicity, even slight changes to methodology, which do not affect chemical compounds or drugs, may affect the testing of NBMs [26, 27]. One such assay however has been well cited as a sensitive and accurate methodology for assessing NBM-induced genotoxicity, the alkaline comet assay [28–30]. The standard version of this assay, the in vivo comet assay, is validated and is an OECD guideline (OECD 489); however, the role of the in vitro comet assay is not currently defined, with efforts currently being made to validate it. In saying this, it is deemed appropriate and recommended under REACH guidelines, already accepted by the European Food Safety Authority (EFSA), and widely used in the screening of cosmetics and pharmaceuticals. The alkaline comet assay is also the most used assay in assessing the genotoxic potential of NBMs [31].

There is a great need and mandatory requirement for assessing the long-term impact of the prolonged exposure and bio-persistence of nanotechnology enabled medical devices and nanomedicines. This has become even more vital as in recent years, new medical device regulations, which came into place in 2020, have been developed. These also require the assessment of long-term exposure. In recent years, various projects and groups have worked tirelessly on bridging the knowledge gaps regarding nanomaterial formulation and toxicity and helping to define and implement appropriate regulatory frameworks for nanomaterials [32]. One such project, NANoREG, supports industry and regulatory authorities in dealing with the safety and environmental implications of engineered nanomaterials [33, 34]. NANoREG focused in particular on emerging materials that would be produced on large scale for manufacturing uses, i.e. TiO_2_, graphene, cellulose, carbon nanotubes, and SiO_2_, nanomaterials which are some of the most commonly used today [35]. PROSAFE, a coordinated support action from the EC, was funded to create a link with industry from NANoREG. Subsequently, to the consolidated data and protocols generated by NANOREG, the risk assessment of commercially viable NBMs adopted for medical devices and ATMPs has been funded by the EC under the BIORIMA project with the clear scope of bridging the knowledge gap. Similarly, to consolidate and integrate the most recent screening test for safety, the REFINE project was also funded by EC with the specific objectives to expand the existing toxicological testing assays as part of the international creation of a regulatory science framework for NBMs. With this in mind, analytical characterisation data and methodological knowledge had to be developed within industrially acceptable standards. This gap has been partially closed by the effort of two key-enabling infrastructures: the European Union Nanomedicine Characterization Laboratory (EUNCL) and the US Nanocharacterization Laboratory (NCI-NCL), whose aligned work supported the translation of nanomaterials from the lab to clinical use by providing detailed and robust assay cascades for the in vitro and in vivo screening of nanomaterials [15]. These assay cascades assist in the determination of nanomaterials ‘reproducibility, safety, and efficacy’, by detailing a set of scientific tests covering pre-screening, physicochemical characterisation, in vitro immunology, haematology, and toxicology [16]. The proof of the success and progress of the NCI-NCL and EUNCL can be seen in their achievement in assisting several nanomaterial formulations in entering and successful progression through clinical trials [36]. The most remarkable success to date has been achieved in the encapsulated-liposome drug delivery systems where there have been several breakthroughs [37–41].

Starting from the extensive lesson learned and read-across developed in this field, a clear objective for engineered materials is to investigate the long-term effect of NBMs for diverse biomedical, cosmetics, and food applications such as liposomes, synthetic polymers, and noble metals. In light of these, this study aims to assess the genotoxic potential of five representative models of NBMs for medicinal applications, to be reflective of the different regulatory testing condition needs. In fact, it is known that a drug delivery system in the form of a bio-persistent metallic nanoparticle (20 nm AuNP), an unloaded polymeric particle (PACA) (as material used as a drug carrier), a drug-loaded PACA particle (i.e. Cabazitaxel, or CBZ), a far-red dye-loaded PACA particle (NR668 PACA), and a dye-loaded liposome (e.g. LipImage™815, a liposome encapsulating IR780 dye, as a medical device for in depth tissue imaging) behave in a completely different way compared to their chemical compound they originate from.

Therefore, the pre-existing knowledge has provided the basis for this study hypothesis since liposomes are regarded as biocompatible and non-genotoxic [42, 43]; nonetheless, literature suggests that they have the potential to mask the toxic effects of some encapsulated materials [44]. Data relating to the genotoxicity of polyacryl-cyanoacrylate (PACA) nanoparticles is also extremely scarce despite being this material an emerging material for advanced drug carrier. The same can be said for both the NR668-loaded and CBZ-loaded PACA, which have not had their genotoxic potential assessed previously. Finally, the most promising inorganic NBM for biomedical application is pegylated gold nanoparticles (AuNPs), since they have been generally regarded and well accepted as a biocompatible material [45, 46]; notwithstanding, they are the least widely studied materials in terms of genotoxicity since up to now they were only used in a single dose exposure. The most recent clinical applications in oncology demand larger doses and multiple administrations, thus extending the exposure time to longer periods [47, 48].

In the perspective of parenteral long-term exposure, the liver (the main organ involved in the filtration of blood) becomes the main point of interaction for administered NBMs; therefore, for this study, human hepatocarcinoma cells (HepG2) were used as an appropriate model [49]. This work was undertaken in parallel with other works undertaken in TCD and within REFINE on the toxicity screening of the same representative panel of NBMs in both HepG2 2D and 3D spheroid models [50]. The main objective behind this interlaboratory comparison work is to assess the reproducibility of the developed protocol for assessing DNA damage induced by NBMs in HepG2 cells, using a modified alkaline comet assay, between two partners within the REFINE project, TCD, Dublin and IBE, Germany.

## Materials and methods

### Chemicals

All chemicals were purchased from Sigma-Aldrich, unless differently specified in the text. Ethyl methanesulfonate (EMS) was purchased from Sigma-Aldrich, Ireland, and used according to manufacturer’s specification. EMS was diluted in cell culture medium and used at a concentration of 15 mM as the genotoxic positive control for experiments.

### NBM preparation

Five different NBMs were used in this study: an IR780 dye-loaded liposome (LipImage™815), poly (ethyl butyl cyanoacrylate) empty nanoparticles (PACA), 20 nm PEG-gold nanoparticles (AuNPs), PACA nanoparticles which encapsulate the far-red dye NR668 (NR668-PACA), and cabazitaxel (CBZ)-loaded PACA nanoparticles (CBZ-PACA).

The AuNPs (Gold Nanospheres, PEG, BioPure™, product number: AUGB20) were purchased commercially from nanoComposix (San Diego, CA) and were provided at a mass concentration of 1.03 mg/ml in ultrapure water (Milli-Q) water mg/ml and a molar particle concentration of 2.5 × 10^8^ particles (mol/l). Particles were 20 nm in size. Dispersion media used was Milli-Q water. The AuNP was deemed sterile and endotoxin free, i.e. BioPure™, with an endotoxin quantity of < 5 EU/ml (within acceptance criteria). AuNP was negatively charged, with a zeta potential of −24 mV. LipImage™815, the IR780 dye-loaded liposome, was kindly provided by CEA-LETI (Grenoble, France) at a particle concentration of 95 mg/ml (9.5%) in 154 mM NaCl and a dye concentration (HPLC) of 239.5 µM (252 µM/100 mg particle). LipImage™815 has a diameter of 80 nm. Dispersion media used was 154 mM NaCl and ascorbic acid (1.75 g/l). LipImage™815 was deemed sterile and endotoxin free, with an endotoxin quantity of < 1UI/ml (within acceptable amount). PACA nanoparticles were provided by SINTEF (Trondheim, Norway) at a concentration of 105 mg/mL in 1 mM HCl in sterile distilled water and were synthesised under aseptic conditions by mini-emulsion polymerisation. Prior to synthesis, all solutions were sterile filtered, and all equipment was autoclaved. An oil phase consisting of poly (ethyl butyl cyanoacrylate) (PEBCA) (Cuantum Medical Cosmetics) containing 2% wt Miglyol 812 (Cremer) and 10 wt% vanillin was prepared. For drug-loaded particles, the oil phase was added 12 wt% cabazitaxel (BioChemPartner), and only 2 wt% of vanillin was used.

For dye-loaded particles, the oil phase was added either 0.4 wt% IR-780-Oleyl (custom synthesis at CEA LETI) or NR668 (modified Nile Red, custom synthesis) [51] at SINTEF. An aqueous phase consisting of 0.1 M HCl containing the two PEG stabilisers (Brij®L23 and Kolliphor®HS15, both Sigma-Aldrich, 5 wt% of each) was added to the oil phase. The water and oil phases were mixed and immediately sonicated for 3 min on ice (6 × 30 s intervals, 60% amplitude, Branson Ultrasonics Digital SONIFIER). The solution was rotated (15 rpm) at a room temperature overnight. The pH was then adjusted to 5.0 to allow further polymerisation for 5 h at room temperature. The dispersions were dialyzed (Spectra/Por dialysis membrane MWCO 100.000 Da) against 1 mM HCl to remove unreacted PEG. The size (z-average), polydispersity index (PDI), and the zeta potential of the NPs in phosphate buffer (10 mM, pH 7.0) were measured by dynamic light scattering (DLS) and laser Doppler micro-electrophoresis using a Zetasizer Nano ZS (Malvern Instruments). To calculate the amount of encapsulated drug, the drug was extracted from the particles by dissolving them in acetone (1:10) and quantified by liquid chromatography coupled to mass spectrometry (LC–MS/MS) using an Agilent 1290 HPLC system coupled to an Agilent 6490 triple quadrupole mass spectrometer.

### NBM characterisation: hydrodynamic diameter and zeta potential measurements of the NPs by NTA and DLS

NTA was undertaken to assess NBM hydrodynamic diameter, using the NS500 Nanosight (Software Version 3.2) (Malvern-Panalytical, UK), according to protocols previously listed by the LBCAM, TCD [52, 53], and which are now well established as validated protocols for NBM under the EUNCL [54, 55].

Nanoparticles were prepared and diluted between 1:5000 and 1:100,000 using D-PBS (-MgCl_2_ and CaCl_2_). A 405-nm laser was used to visualise particles present in a given field of view. Sixty-second recordings of the laser interacting with particles are captured using an EM-CCD camera. The camera level and focus were manually controlled and chosen by the operator (camera level = 10 for the 1:5000 and 1:10,000 dilutions; camera level = 13 for the 1:100,000 dilution). The detection level was chosen by the operator (detection level = 3 in all dilutions), and the recordings were subsequently analysed by the Nanosight 3.2 software to determine particle numbers per frame and sample concentrations. Through the phenomenon of Brownian motion, the particle size can be determined by the software. Thirty-nanometer gold citrate nanoparticles of known size were used as reference materials for the Nanosight.

Dynamic light scattering (DLS) was also undertaken using a Zetasizer Nano ZS system (Malvern UK), running Zetasizer version 7.13, using the EUNCL approved -PCC-001 SOP ‘Measuring Batch Mode DLS’ [56]. 1:100 dilution of the sample for DLS was made up in D-PBS buffer (-MgCl_2_ and CaCl_2_). The sample was pipetted to ensure adequate mixing. The sample was loaded into a DTS0012 disposable cuvette and was subject to a 300-s equilibration time as per the EUNCL SOP. A total of 12 × 10-s runs per measurement were recorded for the sample and were subject to 10 measurements with a 0-s delay between measurements. The backscatter angle (173° NIBS Default) was used in the analysis, with optimum positioning enabled. Automatic attenuation selection was enabled, and the general-purpose analysis mode was chosen.

### Cell culture

Human liver hepatocellular carcinoma (HepG2) cells, supplied by SINTEF, Norway, were used for all experiments in this study. HepG2 cells were maintained in low glucose Dulbecco’s modified Eagle’s medium, supplemented with 10% foetal bovine serum (FBS) and 1% penicillin streptomycin (all Gibco, Invitrogen Ltd, VWR), at 37 °C and 5% CO_2_. For all experiments, passage number restricted to between ten and twenty passages, according to the ATCC supplier. At 80%, confluence cells were detached from T75 flask using TryplE™ (Gibco, Invitrogen, Oregon, USA), centrifuged, and pellet resuspended in 1-mL culture medium. Cells were counted and seeded 1 day prior to nanomaterial treatment at a density of 2.5 × 10^5^ cells in 2.5 mL complete culture medium per well (for each time point).

### Experimental design and exposure endpoints

Different dilutions of each of the NMBs (as characterised above) were freshly resuspended in cell culture media from their stock suspensions at the appropriate time points for mimicking parental administration. To this end, five sublethal concentrations (determined from read across literature) were chosen for the study. Treatment endpoints were over 3 different durations: 30 min, 3 h, and 24 h. From literature, the genotoxic positive control was chosen as 15 mM EMS (ethyl methanesulfonate), a well-established genotoxic agent that induces DNA damage directly [57]. A nanoparticle control, 10 µg/mL TiO_2_ (NM101 supplied by JRC), was also used [58]. Dispersion protocol for TiO_2_ is detailed in a publication by Di Cristo et al. [59]. Each experimental set was undertaken as part of an interlaboratory comparison between partners within the REFINE project, denoted in results by TCD 1 and IBE. To extend the work and further test the robustness of the SOP, a second run of experiments was undertaken on the three primary NBMs (AuNP, LipImage™815, and PACA) by TCD (denoted in results by TCD 2) and further extended with two further PACA formulations, NR668-PACA and CBZ-PACA.

**Fig. 1:**
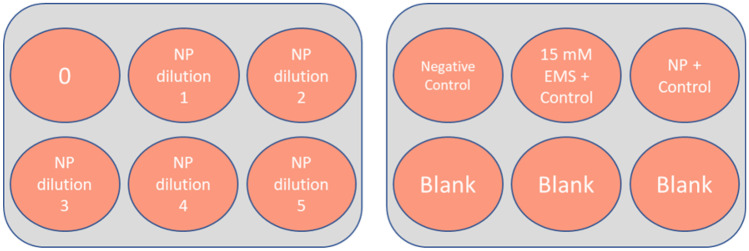
Plate design for alkaline comet assay. Six nanoparticle dilutions and 3 control groups—15 mM EMS positive control, negative control (media), and suitable nanoparticle genotoxic positive control (e.g. 10 µg/mL TiO_2_)

A summary of the concentrations used for each NBMs are reported in Table 1, whereas the experimental plate design for *n* repeats = 3 for statistical relevance is detailed in Fig. 1.

**Table 1:** Nanobiomaterials (NBMs) assessed and their exposure conditions

Nanobiomaterial	Exposure (µg/mL; 30 min, 3 h, 24 h)
AuNP	1, 5, 10, 20, 30
LipImage™815	10, 50, 100, 200, 500
PACA	1, 5, 10, 20, 30
CBZ-PACA	1, 5, 10, 20, 30, 50
NR668-PACA	1, 5, 10, 20, 30, 50

### NBM-customised comet assay

**Fig. 2:**
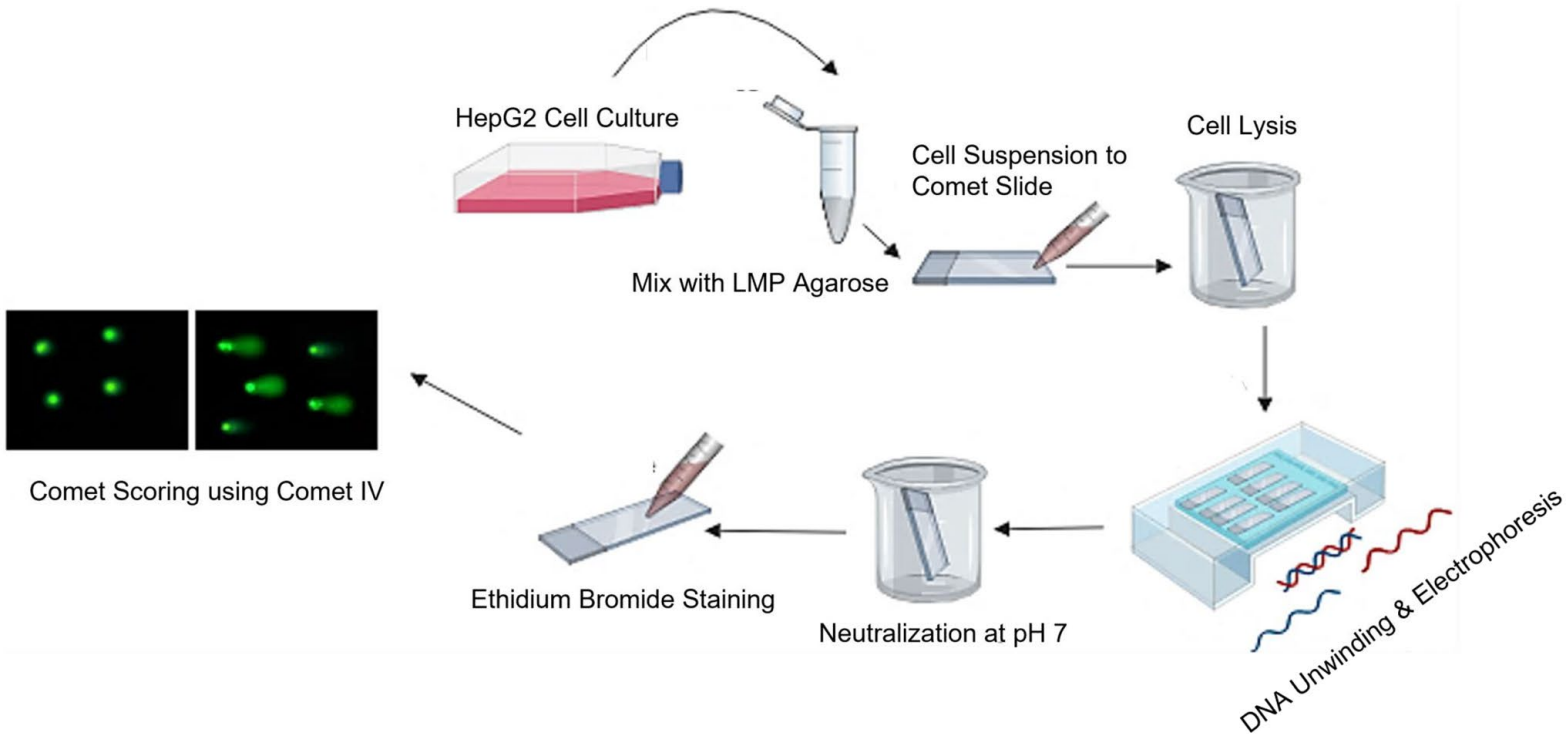
Procedure for the alkaline comet assay

**Fig. 3:**
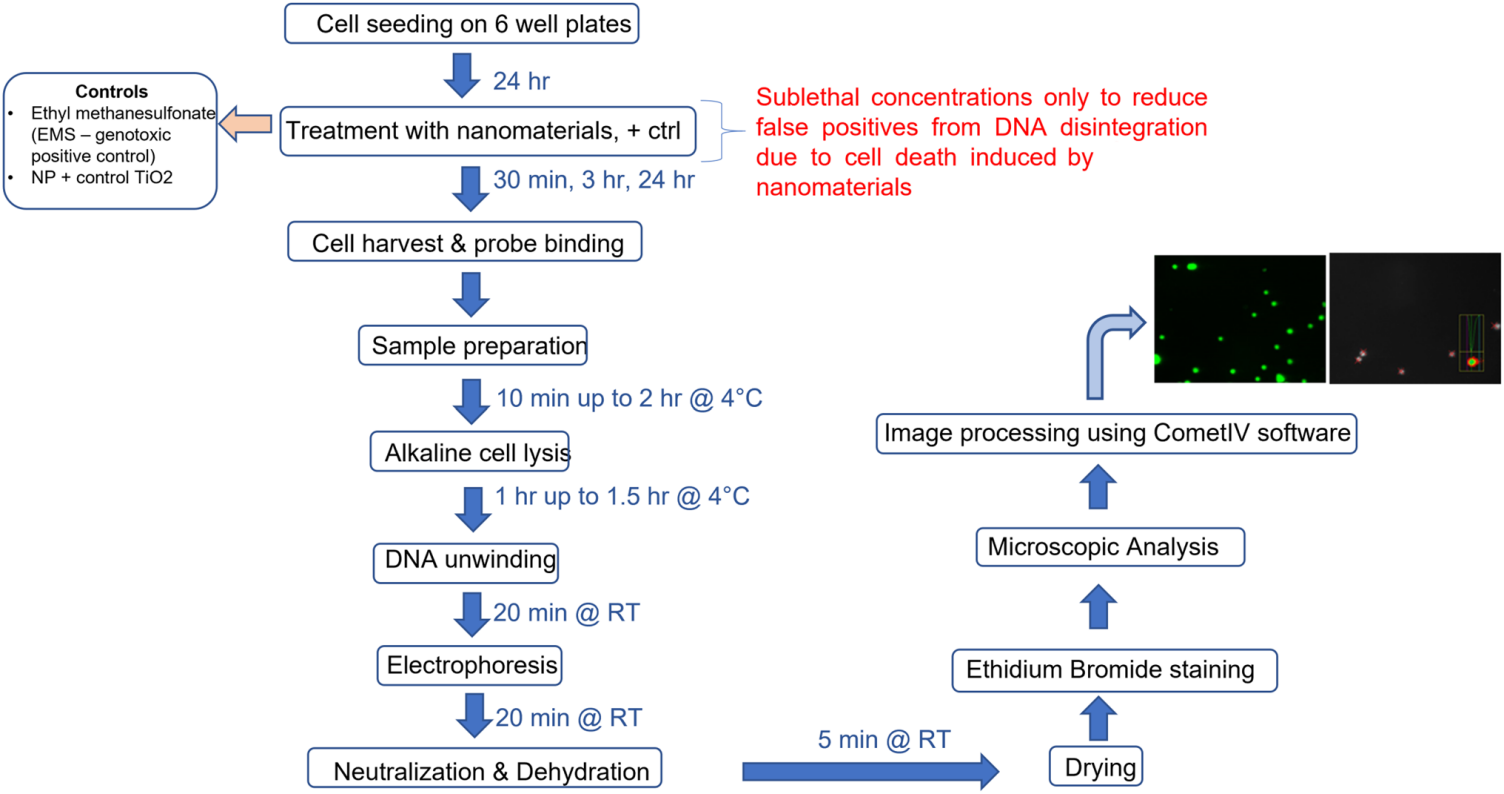
Workflow for alkaline comet assay including image acquisition parameters

The alkaline comet assay protocol for testing NBM genotoxicity was revised from existing OECD guidelines, namely, the Genetic Toxicology Test Guidelines document, as part of the revision discussion process [60]. Extensive discussion on the protocol for this assay was undertaken within EMPA and other European partners as part of the REFINE H2020 project, with the overall aim of establishing a new validated assay for assessing NBM-induced DNA damage. This was based on the single cell gel electrophoresis, or also known as the Comet Assay, which was first detailed in 1988 by Singh et al. [61–63]. Here, it was noted that fragments of damaged DNA migrate from cell, as opposed to undamaged DNA which remains in the nucleus. The resulting pattern resembles a comet-like structure, which contains the intact head of undamaged DNA, and a trailing comet, or tail, of damaged DNA. The extent of the damage can be judged by the intensity or length of the tail [31, 64]. The alkaline comet assay was performed as described in Fig. 2 and in the workflow presented in Fig. 3.

Cells are mixed with LMP agarose, before being cured onto slide and lysed. DNA unwinding and electrophoresis occurs, before neutralisation, staining, and scoring using Comet IV software.

Briefly, 1 day prior to cell harvest, frosted microscope slides were submerged in normal melting point agarose (1.5%) and excess wiped from underside. This was repeated 3 times. Slides were placed horizontally on bench to dry O/N. Thirty minutes before end of culture period, 37.5 µl 1 M of EMS genotoxic positive control was added directly to EMS well. Contents mixed by swirling and plate incubated for a further 30 min. At desired time points, cell culture medium was removed, and cells washed with pre-warmed PBS. 0.2 mL TryplE was added per well and incubated for 5 min. 0.3 mL medium was to neutralise trypsin, and cell suspension was transferred to an Eppendorf and placed on ice. One hundred sixty-microliter low melting point (0.5%) agarose was added to each of 8 labelled Eppendorf’s, and 40 µL of each of the cell suspension added respectively to corresponding Eppendorf. Cells and agarose mixed by carefully pipetting up and down, and 180 µL LMP agarose/cell mixture was pipetted onto the coated superfrost slides. Slide was cover slipped and let cure in fridge for 10 min (or up to 2 h). Cover slip was removed, and slides were placed in precooled lysis solution for1 h. Lysis solution is made as follows: 66.75 ml lysis buffer (see below), 7.5 ml DMSO, and 0.75 Triton X-100. Lysis buffer is made using the following: 2.5 M NaCl, 100 mM EDTA, 10 mM TRIS, and 1% N-lauroylsarcosine sodium salt. Electrophoresis chamber was filled with electrophoresis buffer, made as follows: 75 mL 5 M NaOH and 6.25 mL 0.2 M EDTA, added up to 1.25 L with ddH_2_O. Voltage is fixed at power source to 0.24 V/cm. Current was checked and adjusted to 300 mA by adapting buffer volume. After lysis, slides were taken from solution and drained carefully, before being placed submerged in electrophoresis chamber (ensuring labelled end of slide faces cathode). DNA winding occurred for 20 min without current, before electrophoresis for an additional 20 min. After electrophoresis, slides were removed from chamber, drained, and submerged in TRIS neutralisation buffer for 5 min at RT. The slides were rinsed with ddH2O and then dehydrated by submerging them in absolute ethanol for 5 min at RT. Slides were then dried overnight at RT.

### Image acquisition and data recording

**Fig. 4:**
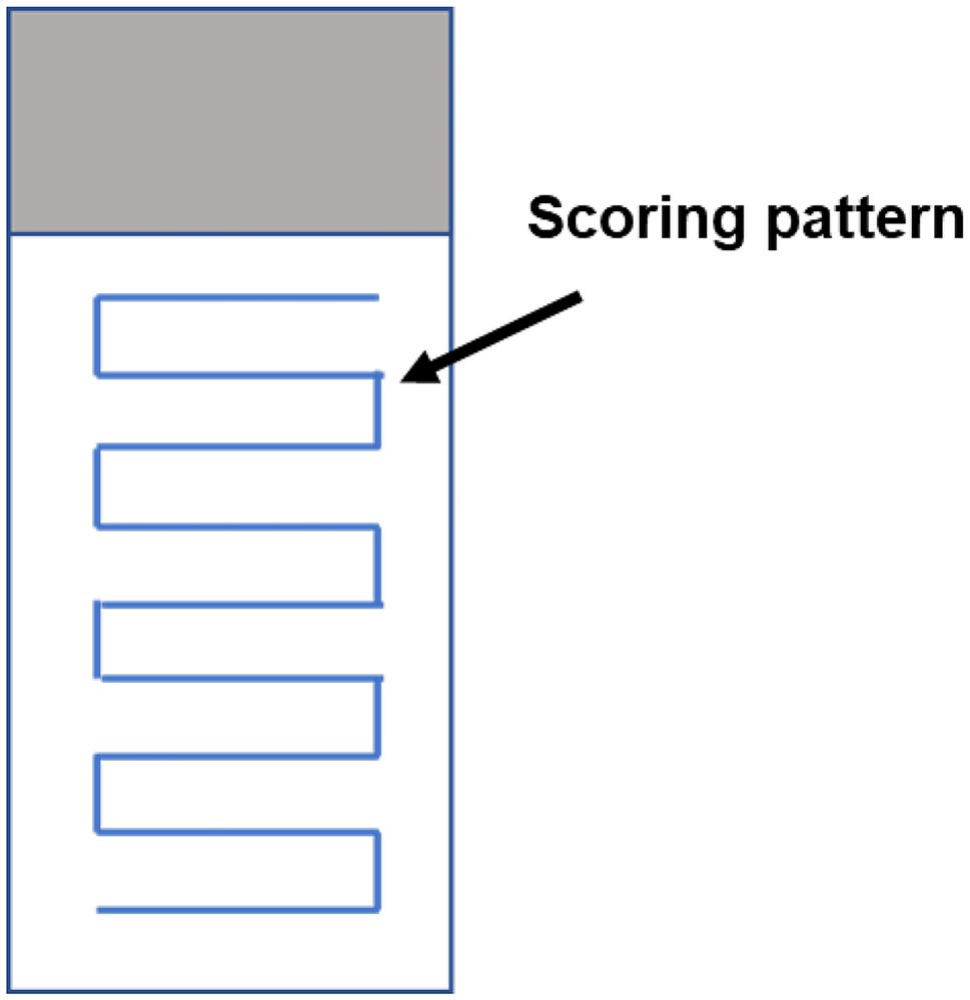
Scoring pattern adopted for the alkaline comet assay. This specific scoring pattern is implemented to avoid re-scoring the same cells/comets and improving the reliability of the data obtained

**Fig. 5:**
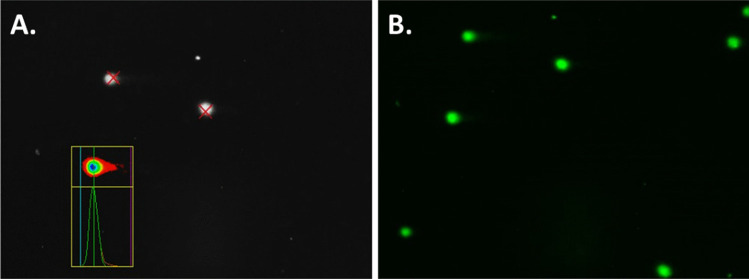
Representative images of scoring following the alkaline comet assay. **A** represents scoring of comets using Comet IV software and **B** represents ethidium bromide-stained cells/comets, processed using ImageJ software

After overnight drying, 60 µl of a 20 µg/mL ethidium bromide solution was added per slide. Cover slip was placed on slide before imaging. The slides were imaged using the specific scoring pattern illustrated in Fig. 4 to ensure that no areas of slides were scored more than once. Slides were imaged using epi-fluorescent microscopy (Nikon TE300) and 10 × objective, with QCapture Software (QImaging tool version 7.0.0.8) to acquire images and Comet IV software (Instem, UK) used to score comets. A representative image of a stained and scored comet slide can be seen in Fig. 5. Comet head and tail are automatically recognised by Comet IV software, and a protected data file is generated. Imaging parameters were kept consistent throughout study (exposure acquisition; 000.154.603 ms; gain 3.870, offset − 1374).

### Quality control and acceptance criteria

One hundred comets must be scored per comet slide using a consistent pattern (illustrated in Fig. 4). The tail % in untreated control must not exceed 10%, in line with the OECD-approved alkaline comet assay protocol [60]. Samples are deemed to not induce genotoxicity if % tail values in NBM-treated samples remain < 10%. Care must be taken to ensure comets are not scored in overconfluent areas of comet slides, and comets on the outer edges of slides must not be scored. ‘Ghost’ or ‘hedgehog’ comets, i.e. comets that only consist of a bright, distinct tail, and no head, must not be scored as these are thought to be indicative of apoptotic cells. Care must be taken to avoid scoring dust and debris. The comet assay is a multi-step protocol, and there is a strong likelihood dust/debris that will build up on slides.

### Statistical analysis

All experiments were replicated in triplicate (*n* = 3) with 100 comets scored per comet slide (*n* = 100). The results are presented as mean values and standard deviation (SD). Two-way ANOVA with Tukey’s multiple comparisons test was undertaken to assess statistical significance of results between each partner. Variation from negative control was tested using Dunnett’s multiple comparisons test. All statistics and graphs are composed using GraphPad Prism software (GraphPad software Inc. Version 9).

## Results

### Characterisation of NPs

The quantitative analysis of the physicochemical characteristics of any NBM is an essential step in order to select a suitable formulation to bring forward in any study. In order to determine if NBMs had remained stable during transit, NBM characterisation was undertaken for the AuNP, LipImage™815, and PACA NBMs prior to any further experimentation, with results compared to characterisation data from supplier.

#### Hydrodynamic diameter and zeta potential

**Fig. 6:**
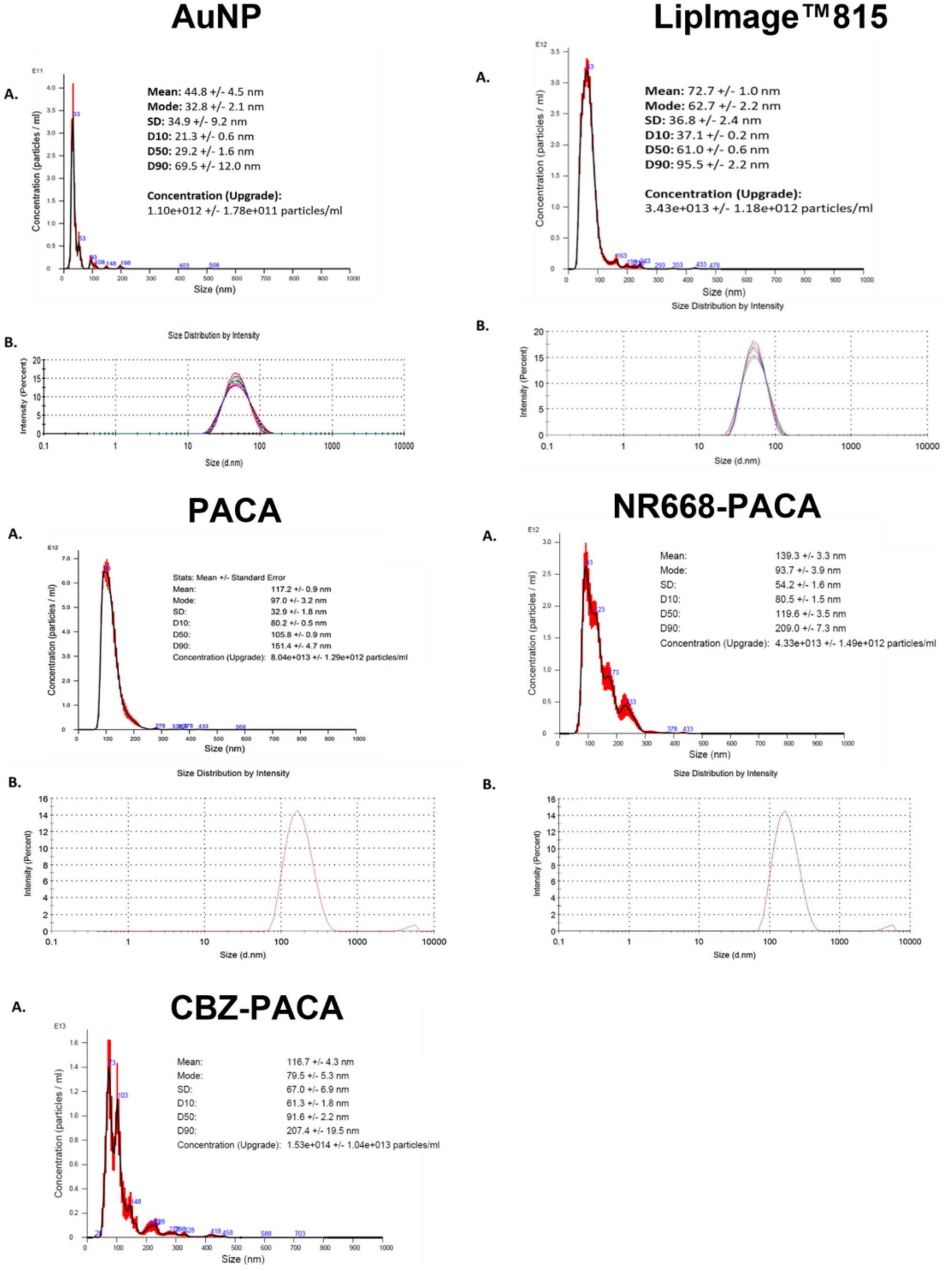
NTA and DLS analysis of each NBM used in this study, namely, AuNP, LipImage™815, PACA, NR668-PACA, and CBZ-PACA. **A** Nanoparticle tracking analysis (NTA) size versus concentration graphs and **B** represents dynamic light scattering (DLS) graphs depicting size distribution and zeta potential at pH 7

NBM hydrodynamic diameter was obtained using NTA analysis, and results obtained were confirmed with measurements obtained using dynamic light scattering (DLS) (Malvern Nano- ZS, Malvern-Panalytical, UK), following the EUNCL protocols previously detailed. Values obtained for both NTA and DLS were compared to datasheets provided by suppliers to assess if NBMs remained stable during transport. NTA and DLS plots for all materials are reported in Fig. 6, with a summary of all characterisation data for each material shown in Table 2.

**Table 2:** Summary of characterisation data by NTA and DLS for each NBM

Readout	AuNP	LipImage™815	PACA	CBZ-PACA	NR668-PACA
DLS	42.45 nm	50.72 nm	134.8	N/A	164.7
NTA	43.4 nm	72.7 nm	94	116.7	140
PDI (DLS)	0.102	0.11	0.092	N/A	0.18
Zeta potential	-26.4 mV	N/A	Not provided	N/A	Not provided

The AuNPs were provided at a mass concentration of 1.00 mg/ml and a molar particle concentration of 2.5 × 10^8^ particles (mol/l) in Milli-Q H_2_O. In line with the EUNCL developed protocols for NBM size and concentration characterisation, analysis was carried out within the LBCAM and compared to supplied data (Supplementary Table 1). NTA reported a mean size of 43.4 nm, which closely resembles the DLS measurements of 42.45 nm. DLS measured an average zeta potential −24 mV at pH 7, making the AuNP anionic. Some small aggregates are present at approximately 100 nm. PDI index, obtained from DLS, is 0.102, making AuNP monodispersed, as shown in Fig. 6. LBCAM characterisation is closely related to supplier values provided.

LipImage™815, the IR780 dye-loaded liposome, was provided by CEA-LETI (France), at a particle concentration of 95 mg/ml (9.5%) and a dye concentration (HPLC) of 239.5 µM (252 µM/100 mg particle). Dispersion media used was 154 mM NaCl and ascorbic acid (1.75 g/l). In line with the EUNCL developed protocols for NBM size and concentration characterisation, analysis was carried out within the LBCAM and compared to supplied data (Supplementary Table 2). NTA reported a mean size of 50.72 nm, with DLS measurements reporting a mean size of 72.7 nm (Table 1). A small aggregate peak is observed at 163 nm (Fig. 6). PDI index, obtained from DLS, is 0.11, making LipImage™815 monodispersed, as shown in Fig. 6. LBCAM characterisation is closely related to supplier values provided.

For the unloaded PACA, NTA and DLS plots are reported in Fig. 6. NTA reported a mean size of 94 nm, and DLS measurement of 134.8 nm. Suppliers noted an average zeta potential −3.2 mV at pH 7, making PACA neutral. PDI index, obtained from DLS, is 0.092, making PACA monodispersed. LBCAM characterisation is closely related to supplier values provided (Supplementary Table 3). The NR 668-loaded PACA NBM was provided at a stock particle concentration of 105 mg NP/ml. Dispersion media used was 1 mM HCl in sterile distilled water. Analysis was carried out within the LBCAM using validated protocols with NTA reporting a mean size of 140 nm and DLS measurement of 164.7 nm (Table 2). DLS measured an average zeta potential −3.6 mV at pH 7, making this PACA NBM neutral. PDI index, obtained from DLS, is 0.18, making PACA monodispersed. LBCAM characterisation is closely related to supplier values provided (Supplementary Fig. 4). The final NBM, CBZ-loaded PACA, was provided at a particle concentration of 107 mg/mL, and with a drug loading concentration of 10.8 (wt% or particle weight as measured by MS) and 12.9 drug concentration in stock (mg/mL), also measured by liquid chromatography mass spectrometry (LC/MS). Characterisation undertaken within LBCAM is compared to supplied data and detailed below (Supplementary Fig. 5). NTA reported a mean size of 116.7 nm. DLS measurement provided by supplier was measured at 121.8 nm and an average zeta potential −5.5 mV at pH 7, making this PACA NBM neutral. PDI index, obtained from DLS, is 0.14.

### DNA strand breaks: the comet assay for assessing in vitro genotoxicity of gold, polymeric, and liposomal nanoparticles

Alkaline comet assay was used to detect DNA strand breaks in HepG2 cells following 30 min, 3 h, and 24 h exposures to six different concentrations of the five NBMs, i.e. AuNP, LipImage™815, unloaded PACA, NR668-PACA, and CBZ-PACA.

#### DNA damage in AuNP-treated HepG2 cells

**Fig. 7:**
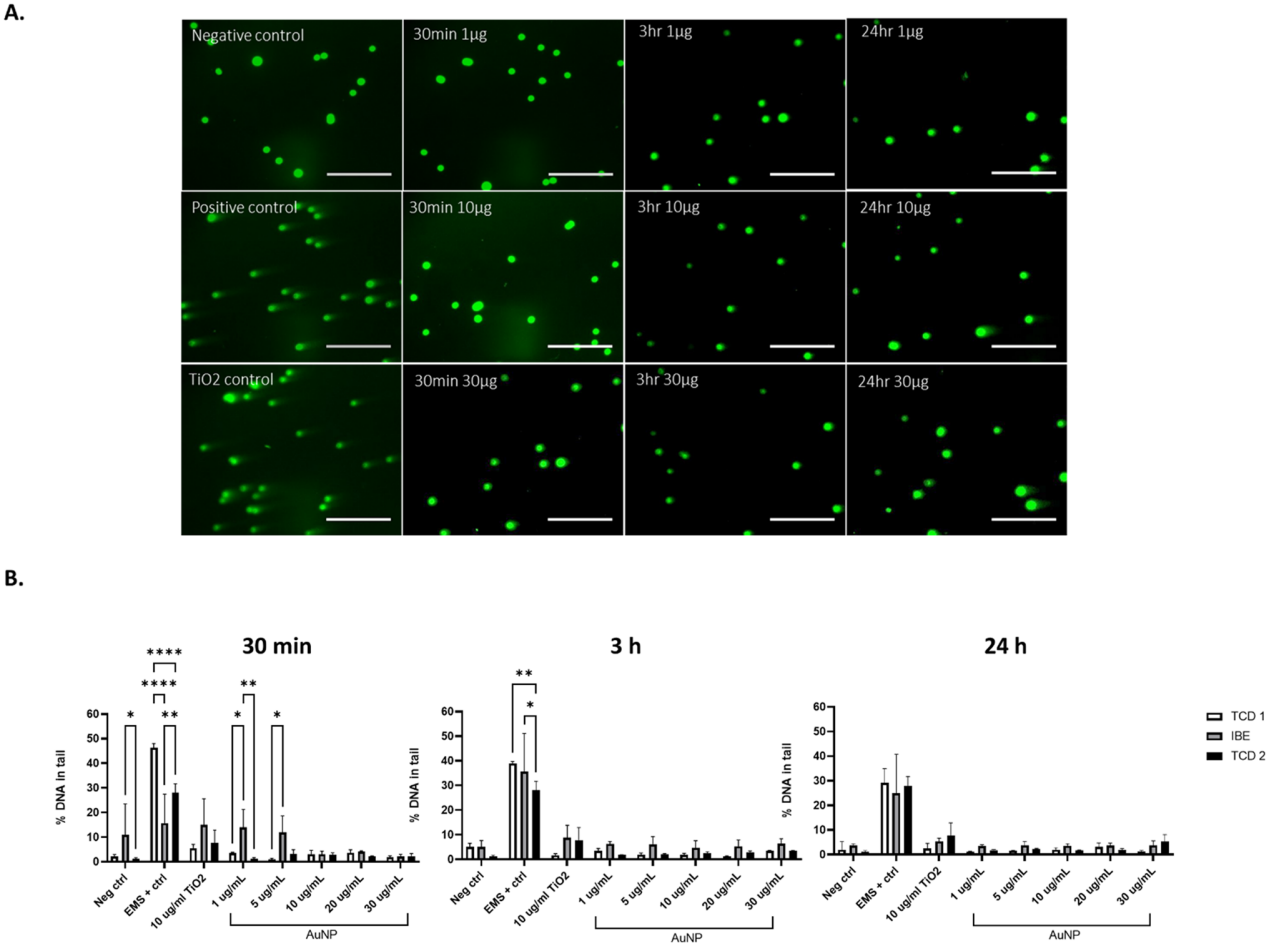
**A** Representative images of ethidium bromide stained HepG2 cells treated with AuNP and appropriate controls for 24 h. **B** Percentage DNA in tail values following treatment with AuNP after (A) 30 min, (B) 3 h, and (C) 24 h. Data representative of a minimum of three independent experiments (*n* = 3), with 100 comets/cells scored per experiment using Comet IV software. Three separate runs undertaken between two partners within REFINE, TCD and IBE, with 2 independent TCD repeats undertaken. Values from one 30-min experiment only (IBE) did not meet acceptance criteria due to an out-of-range negative control (Neg ctrl). Data expressed as mean ± SD. *p < 0.05, **p < 0.05, ****p < 0.0001, statistical analysis by two-way ANOVA with post hoc Tukey test and Dunnett’s multiple comparisons test

HepG2 cells incubated with 20 nm nPEG AuNP caused no notable DNA damage at all concentrations, i.e. 1, 5, 10, 20, and 30 µg/mL, for each time point (Fig. 7), across each of the independent studies between TCD and IBE. All % tail values for each material remain below 5% tail or lower, which is in accordance with acceptance criteria detailed in the ‘Quality control and acceptance criteria’ section, set to be < 10% for tail value. Positive controls (cells treated with 15 mM EMS for 30 min prior to cell lysis) showed increased DNA strand breaks in all experiments. No significant differences were found between and IBE and TCD 1 and 2 treatments after 3-h and 24-h incubations, with significant differences only observed after 1 and 5 µg/mL treatments after 30 min (Supplementary Table 6).

#### DNA damage in LipImage™815-treated HepG2 cells

**Fig. 8:**
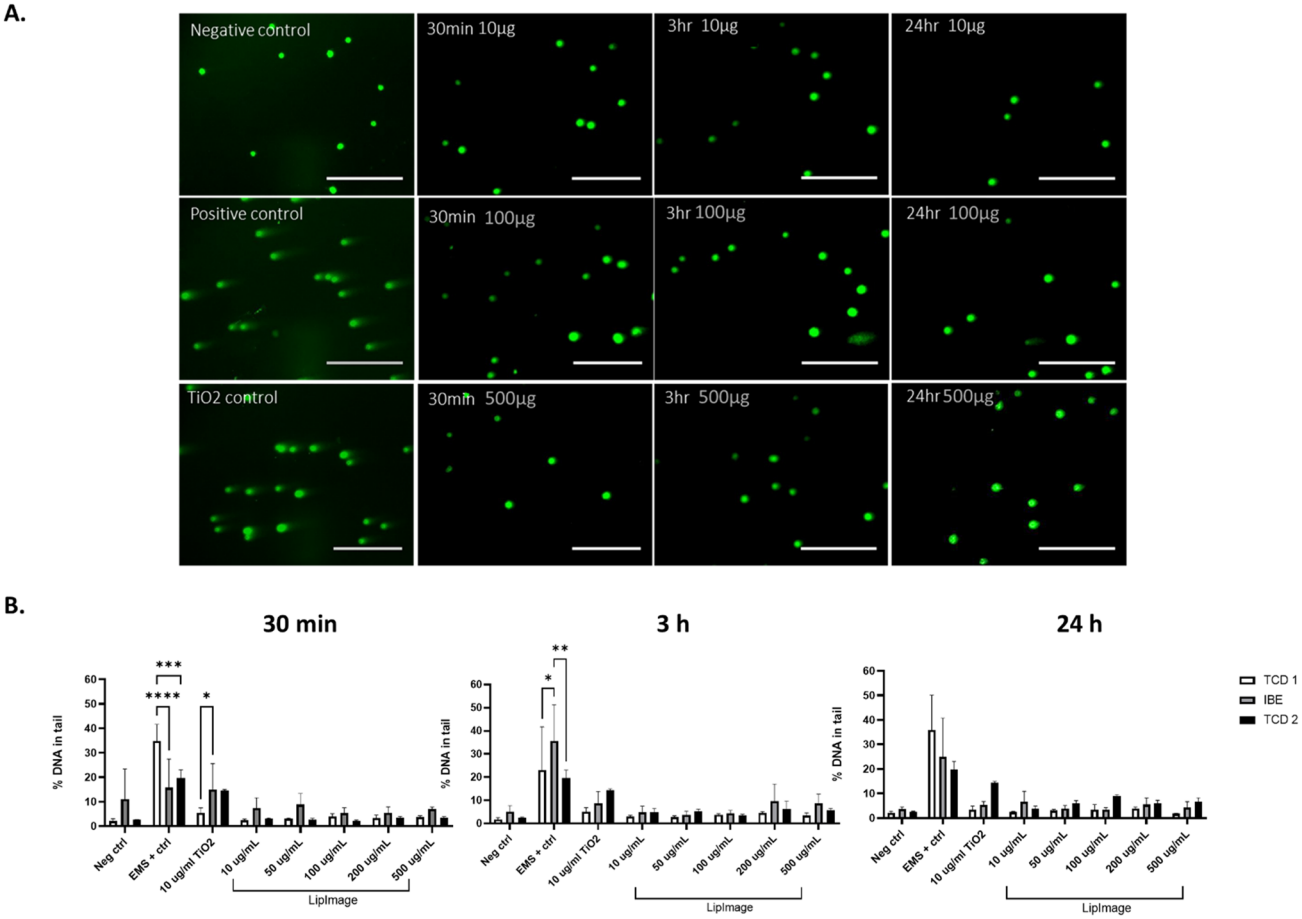
**A** Representative images of ethidium stained HepG2 cells treated with LipImage™815 and appropriate controls for all time points. **B** Percentage DNA in tail values following treatment with LipImage™815 after (A) 30 min, (B) 3 h, and (C) 24 h. Data representative of a minimum of three independent experiments (*n* = 3), with 100 comets scored per experiment using Comet IV software. Three separate runs undertaken between two partners within REFINE, TCD and IBE, with 2 independent TCD repeats undertaken. Values from one 30-min experiment only (IBE) did not meet acceptance criteria due to an out-of-range negative control (Neg ctrl). Data expressed as mean ± SD. *p < 0.05, **p < 0.05, ****p < 0.0001, statistical analysis by two-way ANOVA with post hoc Tukey test Dunnett’s multiple comparisons test

Incubation of HepG2 cell culture with LipImage™815 caused no notable DNA damage at any of the concentrations tested, ranging from 10 to 500 µg/mL across the three independent studies between TCD and IBE partner (Fig. 8). No significant dose-dependent expression of genotoxicity can be observed for any incubations or time points, between the partners and % tail values for all concentrations remain less than 10% DNA damage, which is in accordance with acceptance criteria. However, at least for TCD experiments (1 and 2) concentrations above 10 µg/mL elicited slightly elevated % tail values than the untreated negative control (2.11% tail for Run 1 and 2.6% tail for Run 2 respectively). Any significant differences can be observed in Supplementary Table 7.

#### DNA damage in PACA-treated HepG2 cells

**Fig. 9:**
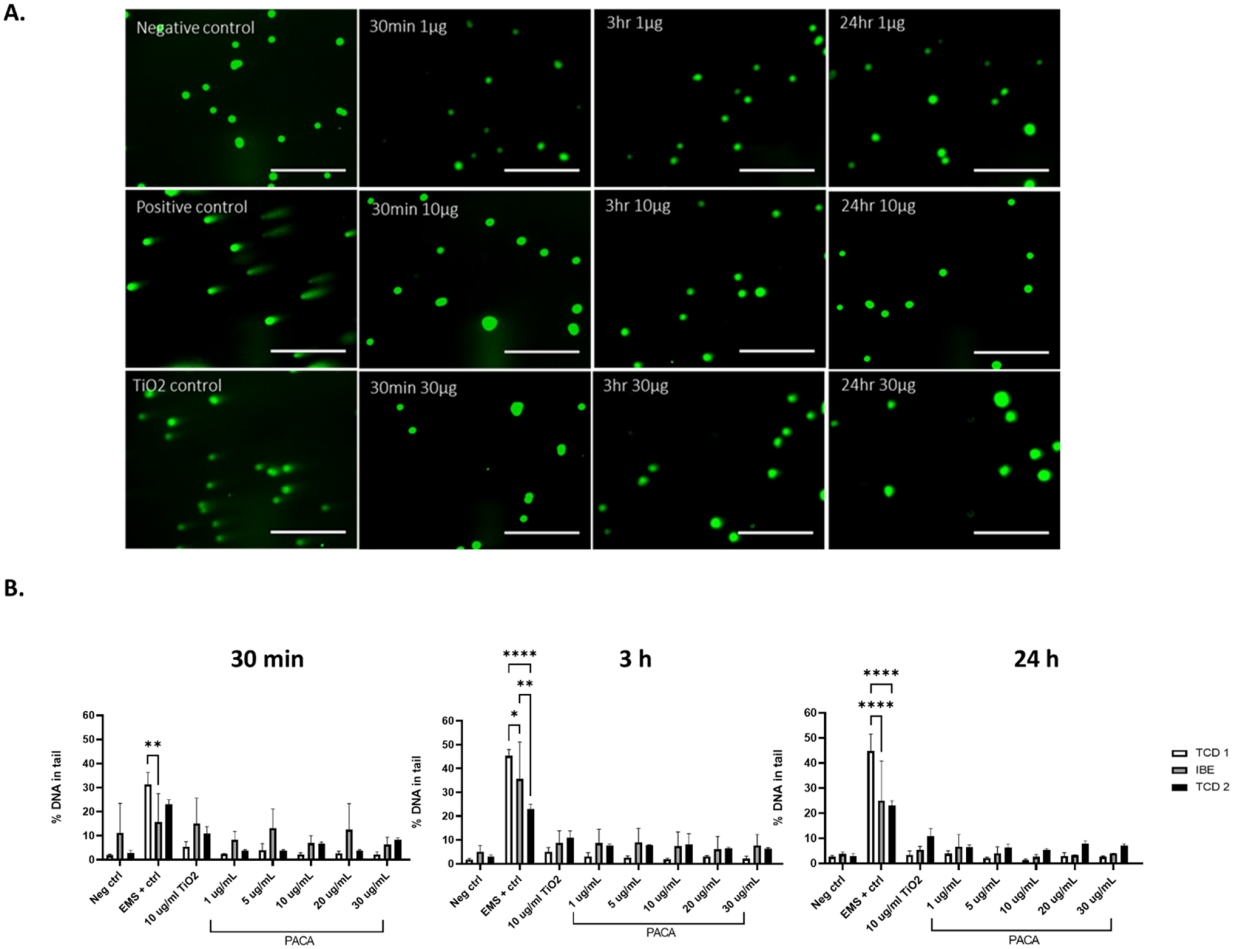
**A** Representative images of ethidium stained HepG2 cells treated with PACA and appropriate controls for all timepoint. **B** Percentage DNA in tail values following treatment with PACA after (A) 30 min, (B) 3 h, and (C) 24 h. Data representative of a minimum of three independent experiments (*n* = 3), with 100 comets scored per experiment using Comet IV software. Three separate runs undertaken between two partners within REFINE, TCD and IBE, with 2 independent TCD repeats undertaken. Values from one 30-min experiment only (IBE) did not meet acceptance criteria due to an out-of-range negative control (Neg ctrl). Data expressed as mean ± SD. *p < 0.05, **p < 0.05, ****p < 0.0001, statistical analysis by two-way ANOVA with post hoc Tukey test Dunnett’s multiple comparisons test

No notable DNA damage was also observed and recorded following treatments with PACA at any of the time points assessed, with all % tail values below the accepted 10% tail value for both sets of experiments undertaken by TCD (Fig. 9). Any significant differences can be observed in Supplementary Table 8.

#### DNA damage in CBZ-loaded and NR668-loaded PACA NBMs

**Fig. 10:**
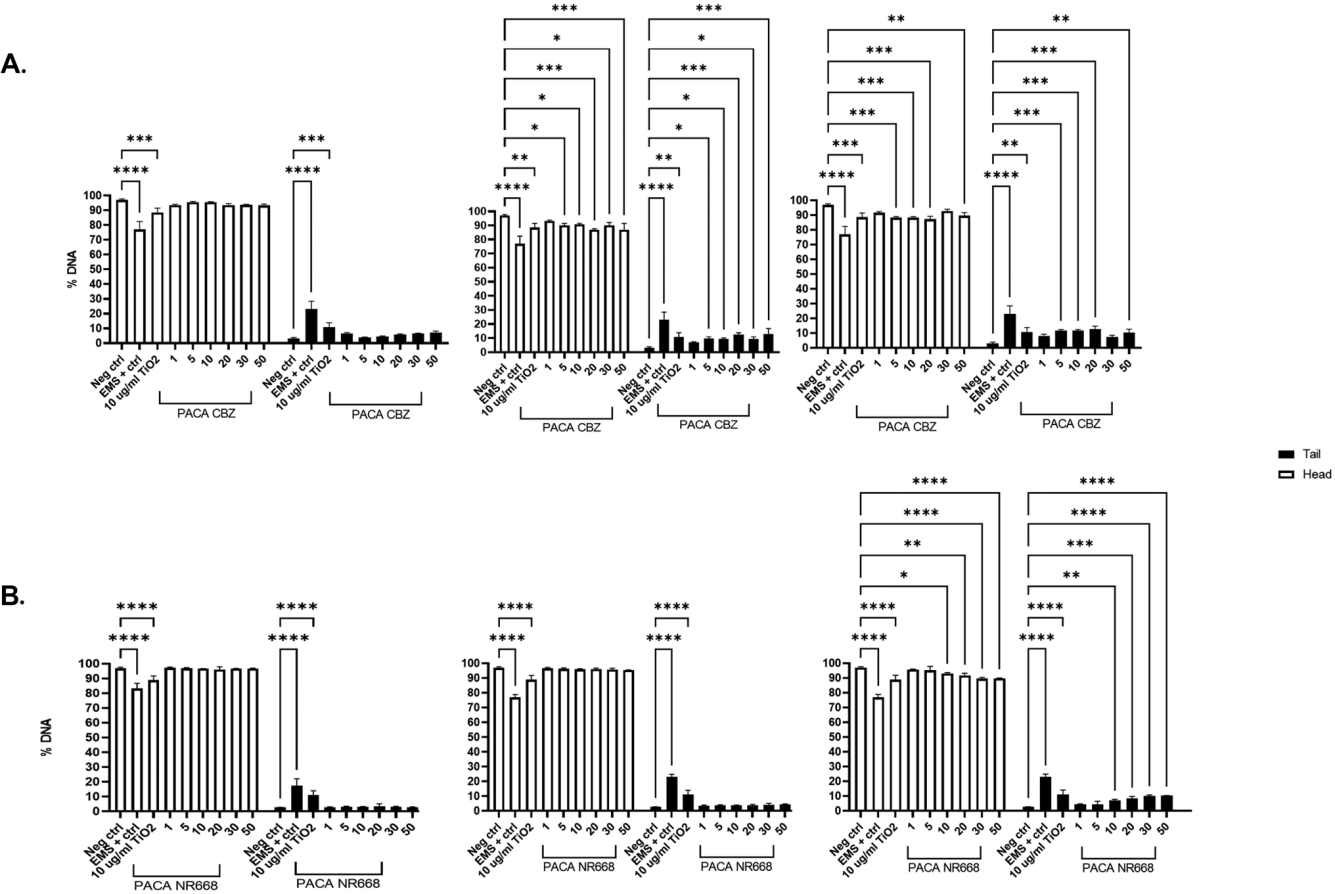
Percentage DNA values following treatment with **A** CBZ-loaded PACA and **B** NR668-loaded PACA. Data representative of a minimum of three independent experiments (*n* = 3), with 100 comets/cells scored per experiment. Data expressed as mean ± SD. Statistical analysis by two-way ANOVA and post hoc Bonferroni test. All graphs and statistical analysis were undertaken using GraphPad Prism 9, Version 9.1.0 (GraphPad Software, Inc., San Diego, USA)

Following on from the assessment of the first three NBMs and once the developed SOP was deemed robust and reproducible, the study was extended by TCD with two further formulations of PACA, NR668-loaded PACA and CBZ-loaded PACA, assessed for their potential to induce DNA damage using the comet assay. CBZ-loaded PACA induced the greatest % DNA damage than any material tested, with % tail values greater than the 10% acceptance criteria for 20 and 30 µg/mL treatments after 3 h and for 5, 10, 20, and 50 µg/mL treatments after 24 h (Fig. 10A). Conversely, NR668 PACA induced no significant DNA damage at any of the concentrations and time points, with % tail values only above the 10% acceptance criteria at 30 and 50 µg/mL after 24 h (10.05 and 10.18% respectively; Fig. 10B).

## Discussion

Whilst there are standardised characterisation methods and toxicity screening assays that exist (including for many areas including cytotoxicity and immunotoxicity), an area which has achieved less attention is genotoxicity screening. It is vitally important to assess the possible genotoxic effect of a NBM prior to a medicinal application or before occupational exposure. Currently, nanomaterial genotoxicity assessment methods are either not fit for purpose (i.e. the bacterial Ames test) or they have been found to interfere with the assay, i.e. the micronucleus test [65, 66]. The alkaline comet assay has proved to be a robust, sensitive, and simple method of determining DNA strand breaks following nanomaterial exposure. Using the alkaline comet assay, it is very possible to have a standardised nanogenotox assay, due to the cell-based nature of the assay and lack of fluorescent readout. In general, the toxicity of nanoparticles depends on their physicochemical properties such as surface charge, particle shape, and size. Therefore, the aim of our study was to investigate the genotoxic effect of a variety of nanoparticle formulations, each of varying composition and size, through an interlaboratory comparison study. Representing metallic carriers used for imaging, drug targeting, and physically triggered treatment was a sterile 20-nm AuNP. To represent liposomal carriers, which can be dye- or drug-loaded and used for imaging and drug targeting, was an IR780-loaded liposome, LipImage™815. Finally, three polymeric NBM formulations were investigated. Huge effort has been placed in recent years on designing NBMs that can increase drug delivery, be more sensitive and more specific for imaging. Poly(alkyl cyanoacrylate), or PACA NBMs, which were first developed and approved as surgical glues, have proven in recent years to be promising drug carriers because of not only their high loading capacity but also their biodegradability [67, 68], and to date many are in late-stage clinical trials. Three PACA formulations were used in this study, an unloaded PACA, a Nile-red (NR668) dye-loaded PACA, and a drug (CBZ)-loaded PACA. This interlaboratory comparison study was undertaken with the view to assess the suitability of the customised alkaline comet assay for the screening of NBM-induced genotoxicity. From a regulatory perspective to date, there are no standardised and validated methods assessing NBM genotoxicity, and this interlaboratory comparison provides an advancement in this area. As most nanoparticles preferentially accumulate in the liver [49, 69], HepG2 cells were chosen as the appropriate cell model for determining DNA damage induced by the NBMs used in this study.

Various studies published up to now have reported genotoxicity of metallic nanoparticles, with different sized AuNPs exhibiting genotoxicity in a size-dependent manner, with only smaller sided AuNPs being active. Xia et al. [70] demonstrated that DNA strand breaks was strongly depended on the size of AuNPs, with larger particles (20 nm and 50 nm) exhibiting no markable DNA damage, whilst smaller AuNPs, i.e. 5 nm, induced dose-dependent DNA damage [70]. This is consistent across other studies demonstrating the genotoxicity of AuNPs [46]. As demonstrated in our study, HepG2 cells incubated with 20-nm AuNP caused no notable DNA damage at all concentrations for each time point. All % tail values remain below 5%, in accordance with acceptance criteria (i.e. < 10% DNA in tail). For 30 min and 24 h time points, tail % values are comparable to that of the negative controls (1.91 ± 0.57% and 1.30 ± 0.39% respectively), possibly due to the antioxidant properties of gold nanoparticles. In terms of reproducibility between partners and both TCD experimental sets, no significant differences were found between IBE and both TCD experimental sets after 3-h and 24-h incubations. Unfortunately, the 30-min control value from IBE did not meet the acceptance criteria (< 10% tail). However, because no such differences were seen between TCD and IBE values after 3 h and 24 h, deviations after 30 min may have been caused by initial handling problems, underpinning that the SOP of the comet assay protocol in itself is robust but requires some training.

Liposomes are generally regarded as non-toxic and biocompatible, and it has been shown that they do in fact mask some of the genotoxic potential of the drug or materials they encapsulate [44]. When incubated with the IR-780 liposome LipImage™815, HepG2 cells show no DNA damage notable (i.e. < 10% tail) at all of the concentrations tested, ranging from 10 to 500 µg/mL. Nevertheless, all concentrations above 10 µg/mL showed slightly higher % DNA tail values than the untreated negative control (1.73–2.45 tail % respectively), although they remained below 10% DNA damage, which is in accordance with acceptance criteria for a non-genotoxic material. LipImage™815 did however show higher % tail values than the AuNP, and also the unloaded PACA presented below. Once again, results from both partners agreed, demonstrating a robust SOP.

Whilst PACA, a polymeric nanoparticle formulation, has shown great promise as a drug carrier for both solid tumours and one which crosses the blood brain barrier, its mode of potential toxicity lacks consistency and has yet to be fully elucidated [71]. There is even less information available on their ability to induce DNA damage. In a similar pattern to the other tested formulations, PACA did not induce significant DNA damage, with all observed % tail values for all concentrations remaining less than 10% DNA damage, in accordance with acceptance criteria. Shorter incubation times (30 min and 3 h) showed higher % tail values than the 24-h incubation time. For all experiments, positive controls (cells treated with 15 mM EMS for 30 min prior to cell lysis) showed increased DNA strand breaks as expected (tail % 29.08–48.98). Higher % tail values were obtained by IBE; however, these were only outside the acceptance criteria for the 5 and 20 µg/mL treatments after 30 min (12.99 and 12.51% respectively).

Following the ILC, TCD experiments were extended by incorporating the other two PACA formulations into the experimental design, CBZ-loaded PACA and NR668-loaded PACA. From all the results presented, CBZ-PACA exhibited the greatest toxicity (CBZ itself has been deemed genotoxic by the EMA). No significant DNA damage was observed following 30-min incubation; however, % DNA damage above the acceptance criteria was observed after 20 and 50 µg/mL treatments, and 5, 10, 20, and 30 µg/mL treatments after 24 h were above acceptance criteria (all other concentrations were close to the threshold). It is possible that this is a consequence of leakage of CBZ from PACA. Nile-red (NR668) is a non-toxic and well-tolerated dye. No DNA damage was observed after 30 min or 3 h following incubation with NR668-loaded PACA, with % DNA in tail values only above the acceptance criteria for the largest concentration, 50 µg/mL, after 24 h.

Although often overlooked in nanomaterial pre-clinical testing, genotoxicity screening is vitally important in assessing whether a material is safe to progress for use in the clinic. Due to prolonged circulation, gradual release, and their need for repeated doses, nanoparticles require more stringent testing methods incorporating both acute and chronic responses. In this interlaboratory comparison study and within the REFINE project, the aim was to test the reproducibility of a standard operating protocol for assessing NBM-induced DNA damage using the alkaline comet assay. Five representative materials were tested, three across two laboratories within the REFINE project, to validate the reproducibility of the SOP, and two more materials to extend the work presented. The data generated from this study not only demonstrates the importance of using dedicated NBM-specific protocols for screening NBMs, but also illustrates the reproducibility of the SOP which was developed by TCD within the regulatory science framework work carried out under the REFINE project. Regarding the genotoxicity screening for NBMs, TCD and IBE have provided significant advances in the area of genotoxicity applied to nanoscale materials, devices, or products.

That being said, there are some limitations to this study. We acknowledge that only one cell line was used for our interlaboratory comparison study, and even though more than one cell line was outside the scope and time constraints of this works, it may be useful to repeat the study with another relevant cell line for robustness. A more significant limitation can be seen in the number of laboratories who took part in the study. Whilst advertised publicly, due to the ongoing COVID pandemic, many labs were either closed or understaffed, and those who did express interest did not have the appropriate equipment/software needed to run the experiments or were unable to obtain the reagents in the allocated time frame. If this study was to be undertaken again, we have no doubt that there would be a greater response to the open call. Addressing these limitations would provide statistical robustness and a stronger confirmation of the results here presented; nonetheless, the value of the study is still great.

## Supplementary Information

Below is the link to the electronic supplementary material.

Supplementary file1 (DOCX 25 KB)

## Data Availability

The data that support the findings of this study are available from the corresponding author, [MAT], on special request.
