# Correction: Evaluating nanobiomaterial-induced DNA strand breaks using the alkaline comet assay

**DOI:** 10.1007/s13346-022-01192-9

**Published:** 2022-06-19

**Authors:** Melissa Anne Tutty, Gabriele Vella, Antje Vennemann, Martin Wiemann, Adriele Prina-Mello

**Affiliations:** 1 Nanomedicine and Molecular Imaging Group, Clinical Medicine, Trinity Translational Medicine Institute (TTMI), Trinity College Dublin, St James’s Hospital, Dublin 8, Ireland; 2 Laboratory for Biological Characterization of Advanced Materials (LBCAM), TTMI, Trinity College Dublin, St James’s Hospital, Dublin 8, Ireland; 3 IBE R&D Institute for Lung Health gGmbH, Münster, Germany; 4 Trinity St James’s Cancer Institute, Trinity College Dublin, St James’s Hospital, Dublin 8, Ireland

## Correction to: Drug Delivery and Translational Research https://doi.org/10.1007/s13346-022-01178-7

In the original online version of this article Gabriele Vella's given name is misspelled. The original article was corrected.

