# Supplementary material for: Evaluating nanobiomaterial-induced DNA strand breaks using the alkaline comet assay
Source: Drug Deliv Transl Res. 2022 May 25;12(9):2243–58. doi: 10.1007/s13346-022-01178-7 (PMC9360128; doi:10.1007/s13346-022-01178-7)
Supplement: Supplementary file 1 — Supplementary file1 (DOCX 25 KB) [file 13346_2022_1178_MOESM1_ESM.docx]

# Supplementary Information DDTR-D-22-00018

**Supplementary Table 1**. Summary and comparison of characterization data by NTA and DLS for AuNP

| **AuNP Measured Parameter** | **Supplier Value** | **TCD Value** |
| --- | --- | --- |
| Mean hydrodynamic size (DLS) | 45.5 nm | 42.45 nm |
| Mean hydrodynamic size (NTA) | Not provided | 43.4 nm |
| Polydispersity Index (PDI) (DLS) | Not provided | 0.102 |
| Zeta Potential | -26 mV | -26.4 mV |

**Supplementary Table 2.** Summary and comparison of characterization data by NTA and DLS for LipImage™815

| **LipImage™815** | **Supplier Value** | **TCD Value** |
| --- | --- | --- |
| Mean hydrodynamic size  (DLS) | 52.2 nm | 50.72 nm |
| Mean hydrodynamic size (NTA) | Not provided | 72.7 nm |
| Polydispersity Index (PDI) (DLS) | <0.102 | 0.11 |

**Supplementary Table 3.** Summary and comparison of characterization data by NTA and DLS for unloaded PACA

| **PACA Measured Parameter** | **Supplier Value** | **TCD Value** |
| --- | --- | --- |
| Mean hydrodynamic size (DLS) | 134 | 134.8 |
| Mean hydrodynamic size (NTA) | Not provided | 94 |
| Polydispersity Index (PDI) (DLS) | 0.11 | 0.092 |
| Zeta Potential | -3.2 mV | Not provided |

**Supplementary Table 4.** Summary and comparison of characterization data by NTA and DLS for NR668-PACA

| **PACA NR668 Measured Parameter** | **Supplier Value** | **TCD Value** |
| --- | --- | --- |
| Mean hydrodynamic size (DLS) | 178 | 164.7 |
| Mean hydrodynamic size (NTA) | Not provided | 140 |
| Polydispersity Index (PDI) (DLS) | 0.28 | 0.18 |
| Zeta Potential | -3.6 mV | Not provided |

**Supplementary Table 5.** Summary and comparison of characterization data by NTA and DLS for CBZ-PACA

| **CBZ-PACA Measured Parameter** | **Supplier Value** | **TCD Value** |
| --- | --- | --- |
| Mean hydrodynamic size (DLS) | 121.8 | N/A |
| Mean hydrodynamic size (NTA) | Not provided | 116.7 |
| Polydispersity Index (PDI) (DLS) | 0.14 | N/A |
| Zeta Potential | -5.5 mV | N/A |

**Supplementary Table 6.** Significant differences between negative control and test concentrations; AuNP experiments

| **Labs** | | **NT** | **EMS** | **TiO2** | **1** | **5** | **10** | **20** | **30** |
| --- | --- | --- | --- | --- | --- | --- | --- | --- | --- |
| **30 min** | **TCD 1** | ns | **** | ns | ns | ns | ns | ns | ns |
|  | **IBE** | ns | **ns** | ns | ns | ns | ns | ns | ns |
|  | **TCD 2** | ns | **** | ns | ns | ns | ns | ns | ns |
| **3 h** | **TCD 1** | ns | **** | ns | ns | ns | ns | ns | ns |
|  | **IBE** | ns | **** | ns | ns | ns | ns | ns | ns |
|  | **TCD 2** | ns | **** | ns | ns | ns | ns | ns | ns |
| **24 h** | **TCD 1** | ns | **** | ns | ns | ns | ns | ns | ns |
|  | **IBE** | ns | **** | ns | ns | ns | ns | ns | ns |
|  | **TCD 2** | ns | **** | ns | ns | ns | ns | ns | ns |

**Supplementary Table 7.** Significant differences between negative control and test concentrations; LipImage™815 experiments

|  | | **NT** | **EMS** | **TiO2** | **10** | **50** | **100** | **200** | **500** |
| --- | --- | --- | --- | --- | --- | --- | --- | --- | --- |
| **30 min** | **TCD 1** | ns | **** | ns | ns | ns | ns | ns | ns |
|  | **IBE** | ns | ns | ns | ns | ns | ns | ns | ns |
|  | **TCD 2** | ns | **** | * | ns | ns | ns | ns | ns |
| **3 h** | **TCD 1** | ns | **** | ns | ns | ns | ns | ns | ns |
|  | **IBE** | ns | **** | ns | ns | ns | ns | ns | ns |
|  | **TCD 2** | ns | **** | ns | ns | ns | ns | ns | ns |
| **24 h** | **TCD 1** | ns | **** | ns | ns | ns | ns | ns | ns |
|  | **IBE** | ns | **** | ns | ns | ns | ns | ns | ns |
|  | **TCD 2** | ns | **** | * | ns | ns | ns | ns | ns |

**Supplementary Table 8.** Significant differences between negative control and test concentrations; PACA experiments

|  | | **NT** | **EMS** | **TiO2** | **10** | **50** | **100** | **200** | **500** |
| --- | --- | --- | --- | --- | --- | --- | --- | --- | --- |
| **30 min** | **TCD 1** | ns | **** | ns | ns | ns | ns | ns | ns |
|  | **IBE** | ns | ns | ns | ns | ns | ns | ns | ns |
|  | **TCD 2** | ns | *** | ns | ns | ns | ns | ns | ns |
| **3 h** | **TCD 1** | ns | **** | ns | ns | ns | ns | ns | ns |
|  | **IBE** | ns | **** | ns | ns | ns | ns | ns | ns |
|  | **TCD 2** | ns | **** | ns | ns | ns | ns | ns | ns |
| **24 h** | **TCD 1** | ns | **** | ns | ns | ns | ns | ns | ns |
|  | **IBE** | ns | **** | ns | ns | ns | ns | ns | ns |
|  | **TCD 2** | ns | **** | ns | ns | ns | ns | ns | ns |
